# Efficacy of Thromboprophylaxis in Preventing Thrombotic Events in Pediatric Patients With COVID-19 or Multisystem Inflammatory Syndrome: A Systematic Review

**DOI:** 10.7759/cureus.80002

**Published:** 2025-03-03

**Authors:** Jaqueline L Castillo, Andrea Medel Sánchez, Danna M Miranda Lugo, Natalia Núñez Muratalla, Cristina P Agredano Chávez, Juan M Cervantes Carrillo, Gabriela V Martínez Sánchez, Mildred Rios Torres, Karina Aguilar García, Lilia Rubio Alfaro, Mauricio Montelongo Quevedo, Jose R Flores Valdés

**Affiliations:** 1 General Medicine, Universidad Autónoma de Guadalajara, Guadalajara, MEX; 2 General Practice, Universidad de Guanajuato, León, MEX; 3 General Practice, Universidad Nacional Autónoma de México, Mexico City, MEX; 4 General Medicine, Universidad de Colima, Colima, MEX; 5 General Practice, Benemérita Universidad Autónoma de Puebla, Puebla, MEX; 6 General Medicine, Tecnológico de Monterrey, Mexico City, MEX; 7 General Medicine, Universidad Autónoma de Chihuahua, Chihuahua, MEX; 8 General Practice, Oncology Consultants PA, Houston, USA

**Keywords:** covid-19, mis-c, pediatric, thromboprophylaxis, thrombosis

## Abstract

The COVID-19 pandemic has been associated with a broad spectrum of clinical manifestations, including multisystem inflammatory syndrome in children (MIS-C), a rare but serious condition characterized by a proinflammatory and hypercoagulable state. MIS-C has been linked to an elevated risk of venous thromboembolism (VTE), necessitating a focus on thromboprophylaxis to prevent potentially fatal complications in pediatric patients. This systematic review aims to evaluate the association between COVID-19/MIS-C and thromboembolism and to assess the efficacy of thromboprophylaxis protocols in reducing thrombotic events and mortality in children. A systematic review was conducted following the Preferred Reporting Items for Systematic Reviews and Meta-Analyses (PRISMA) 2020 guidelines. Literature searches were performed in PubMed, Cochrane, and Science Direct databases. Randomized controlled trials, cohort studies, and case-control studies reporting on thromboprophylaxis, thrombotic events, and associated outcomes in pediatric patients (<21 years) with COVID-19 and/or MIS-C were included. The Newcastle-Ottawa Scale was used to assess the quality of included studies. Primary outcomes were the incidence of thrombotic events and mortality, while secondary outcomes included bleeding events, clinical recovery, and changes in coagulation markers. Of the 375 articles identified, three studies (n=771 patients) met the inclusion criteria. Thromboprophylaxis protocols primarily included low molecular weight heparin (LMWH) such as enoxaparin and antiplatelet agents such as aspirin, with varied doses and treatment durations. Thrombotic events were reported in 3.3% of patients, with a higher incidence in MIS-C cases compared to COVID-19 alone. Prophylactic anticoagulation was effective in preventing thrombotic events in high-risk patients without increasing the risk of major bleeding. The studies emphasized individualized treatment approaches based on risk factors such as elevated D-dimer levels, obesity, prolonged immobilization, and central venous catheter presence. All studies reported a low mortality rate, ranging from 0% to 2.2%, highlighting the potential benefit of thromboprophylaxis in this population. Pediatric patients with MIS-C or severe COVID-19 are at an increased risk of thrombotic complications due to their heightened proinflammatory and hypercoagulable states. Thromboprophylaxis using enoxaparin and aspirin appears effective in reducing thrombotic events and mortality in these patients. Individualized protocols based on clinical risk factors and D-dimer levels are critical to optimizing outcomes while minimizing bleeding risks. Standardized, evidence-based guidelines are needed to refine thromboprophylaxis strategies and determine the optimal duration of therapy in this vulnerable population. Further research is essential to better understand the role of coagulation markers in guiding treatment cessation and improving outcomes.

## Introduction and background

Between 2020 and early 2021, the severe acute respiratory syndrome coronavirus 2 (SARS-CoV-2) rapidly spread worldwide, causing the COVID-19 pandemic [1]. Multisystem inflammatory syndrome in children (MIS-C) was first detected in April 2020 and is currently linked to COVID-19. MIS-C is defined by multiorgan involvement, laboratory markers of inflammation, fever, severe illness, and evidence of a recent SARS-CoV-2 infection [2]. The clinical spectrum in children ranges from persistent fever and inflammation to characteristic features of Kawasaki-like disease, shock, multiple organ failure, and death in severely ill patients. Most children who contract the SARS-CoV-2 virus only have mild illness.

While coagulation and inflammation are increased in COVID-19 infection, MIS-C is characterized by a strong proinflammatory and procoagulant state. It is increasingly evident that this pediatric condition is related to thrombotic events [1]. On the other hand, venous thromboembolism (VTE) is the obstruction of any deep vein by a blood clot, including pulmonary embolism (PE) or deep vein thrombosis (DVT) and can occur spontaneously. The pathophysiology of venous thrombosis is described by Virchow's Triad as hypercoagulability, hemodynamic changes (as stasis or turbulence) and endothelial injury [1,3]. As the pandemic progressed, reports surfaced indicating elevated rates of venous and arterial thrombotic events in children affected by COVID-19 and MIS-C emerged [2].

Several studies have investigated VTE in COVID-19 patients. Some literature discusses the need for a better understanding of COVID-19 and its clinical course as a complication of the disease [1]. This is significant because initial studies have identified alarmingly high rates of PE in patients with severe COVID-19 who were treated in intensive care units (ICUs), with reported VTE incidences of up to 50% [4]. Emerging data among hospitalized children with COVID-19 and MIS-C confirms the presence of a prothrombotic state. These patients have an increased rate of thrombosis and mortality, specifically among those with clinical thrombosis, supporting the need for thromboprophylaxis. Furthermore, patients with MIS-C are at the highest risk of thrombosis (6.5%, 13 times higher than baseline) followed by COVID-19 (2.1%, four times higher than baseline) [5].

In response to the clinical challenges and the absence of high-quality evidence, expert groups and scientific societies have conducted several experimental studies to generate guidance statements addressing questions concerning the diagnosis, prevention, and treatment of VTE in patients with COVID-19. These studies suggest the broad application of thromboprophylaxis in pediatric patients with severe COVID-19 without high bleeding risk [4].

Experts recommend maintaining a low threshold for initiating thromboprophylaxis in pediatric patients, such as the presence of a single additional prothrombotic risk factor or significantly elevated plasma D-dimer levels - five or more times the upper limit of normal. Some institutions have developed thromboprophylaxis protocols informed by local experience, using elevated D-dimer levels to guide decisions, despite limited evidence on the utility of D-dimer in assessing the severity of prothrombotic states in children. Additionally, concerns persist regarding the effectiveness of prophylaxis-intensity regimens in critically ill pediatric patients with COVID-19.

Considering the background and the complications associated with this syndrome, the primary objective of this systematic review is to identify studies that report the association between COVID-19 and/or MIS-C thromboembolism, as well as the effects of thromboprophylaxis on these events, particularly in pediatric patients. We aim to review different thromboprophylaxis protocols, comparing drugs used, their dosages, and the duration of interventions to determine which protocol is associated with the lowest rates of thrombotic events and mortality.

## Review

Methods 

This study followed the Preferred Reporting Items for Systematic Reviews (PRISMA) 2020 guidelines and principles of evidence-based medicine to ensure a thorough and systematic approach to our review [6,7].

Search Methods

Stringent criteria were established to include only high-quality studies. The exclusion criteria were rigorously applied to maintain the quality and relevance of the studies analyzed. Studies that did not focus on pediatric populations dealing with thrombotic events due to MIS-C or COVID-19, as well as those that did not include the thromboprophylaxis methods, were excluded. Additionally, studies that were unavailable in full text or could not be obtained via interlibrary loans were excluded. The inclusion criteria were designed to establish a robust and high-quality evidence base, focusing mainly on reviewing randomized controlled trials, cohort studies, and case-control studies. The study was registered with PROSPERO (International Prospective Register of Systematic Reviews) under the registration number CRD42024588449. The literature search was conducted across multiple databases: PubMed, Cochrane, and Science Direct (Table 1). The search strategy employed Medical Subject Headings (MESH) terms and free-text terms relevant to our research question. The article selection process was guided by a PRISMA flowchart. This meticulous approach enabled the creation of a homogeneous dataset, facilitating a more accurate and reliable analysis of the results. Only peer-reviewed journal articles published from the year 2020 to the present were included in the review. The search strategy was formulated using a combination of keywords for each database, employing the Boolean operators "OR" and "AND" as detailed in Table 1. Only manuscripts written in English and Spanish were considered. Three reviewers independently evaluated articles for initial inclusion based on title and abstract. Full texts were subsequently retrieved and assessed for eligibility.

**Table 1:** Search Strategy for Each Database and Registry

Database	Search	Results
Pubmed	("child"[MeSH Terms] OR "adolescent"[MeSH Terms] OR "adolescent"[MeSH Terms] OR ("child"[MeSH Terms] OR "child"[All Fields] OR "children"[All Fields] OR "child*"[All Fields] OR "children"[All Fields] OR "child"[All Fields]) OR ("adolescency"[All Fields] OR "adolescent"[MeSH Terms] OR "adolescent"[All Fields] OR ("infant"[MeSH Terms] OR "infant"[All Fields] OR "infants"[All Fields] OR "infant*"[All Fields]) OR "infant"[MeSH Terms])) AND ((("covid 19"[All Fields] OR "covid 19"[MeSH Terms] OR "covid 19 vaccines"[All Fields] OR "covid 19 vaccines"[MeSH Terms] OR "covid 19 serotherapy"[All Fields] OR "covid 19 nucleic acid testing"[All Fields] OR "covid 19 nucleic acid testing"[MeSH Terms] OR "covid 19 serological testing"[All Fields] OR "covid 19 serological testing"[MeSH Terms] OR "covid 19 testing"[All Fields] OR "covid 19 testing"[MeSH Terms] OR "sars cov 2"[All Fields] OR "sars cov 2"[MeSH Terms] OR "severe acute respiratory syndrome coronavirus 2"[All Fields] OR "ncov"[All Fields] OR "2019 ncov"[All Fields] OR (("coronavirus"[MeSH Terms] OR "coronavirus"[All Fields] OR "cov"[All Fields]) AND 2019/11/01:3000/12/31[Date - Publication]) OR ("sars cov 2"[MeSH Terms] OR "sars cov 2"[All Fields] OR "covid"[All Fields] OR "covid 19"[MeSH Terms] OR "covid 19"[All Fields]) OR ("sars cov 2"[MeSH Terms] OR "covid 19"[MeSH Terms]) OR "covid 19"[MeSH Terms]) AND (("multisystem"[All Fields] OR "multisystemic"[All Fields] OR "multisystems"[All Fields]) AND ("inflammatories"[All Fields] OR "inflammatory"[All Fields]) AND ("syndrom"[All Fields] OR "syndromal"[All Fields] OR "syndromally"[All Fields] OR "syndrome"[MeSH Terms] OR "syndrome"[All Fields] OR "syndromes"[All Fields] OR "syndrome s"[All Fields] OR "syndromic"[All Fields] OR "syndroms"[All Fields]))) OR (("multisystem"[All Fields] OR "multisystemic"[All Fields] OR "multisystems"[All Fields]) AND ("inflammatories"[All Fields] OR "inflammatory"[All Fields]) AND "syndrome"[MeSH Terms])) AND ("aspirin"[MeSH Terms] OR "aspirin"[All Fields] OR "aspirins"[All Fields] OR "aspirin s"[All Fields] OR "aspirine"[All Fields] OR "aspirin"[MeSH Terms] OR "thromboprophylaxis"[All Fields]) AND (("clinical"[Title/Abstract] AND "trial"[Title/Abstract]) OR "clinical trials as topic"[MeSH Terms] OR "clinical trial"[Publication Type] OR "random*"[Title/Abstract] OR "random allocation"[MeSH Terms] OR "therapeutic use"[MeSH Subheading] OR ("cohort studies"[MeSH Terms] OR "case-control studies"[MeSH Terms] OR "comparative study"[Publication Type] OR "risk factors"[MeSH Terms] OR "cohort"[Text Word] OR "compared"[Text Word] OR "groups"[Text Word] OR "case control"[Text Word] OR "multivariate"[Text Word]))	57
Cochrane	#1 pediatrics	32312
	#2 child	211452
	#3 adolescent	165760
	#4 #1 OR #2 OR #3	322931
	#5 inflammatory syndrome	10048
	#6 #4 AND #5	1821
	#7 Anticoagulants	9017
	#8 #6 AND #7	51
Science Direct	pediatrics AND inflammatory syndrome AND Thromboprophylaxis	267

Selection Criteria

Types of participants: This study established selection criteria that included only pediatric patients <21 years of age who were hospitalized for symptomatic COVID-19 or were diagnosed with MIS-C and who received thromboprophylaxis. MIS-C diagnosis must be made with the symptomatic presentation (persistent fever, gastrointestinal symptoms, rash, conjunctivitis, mucous membrane involvement, red or swollen lips, strawberry tongue, neurocognitive symptoms, respiratory symptoms, lymphadenopathy) and/or clinical findings of shock; criteria met for complete Kawasaki disease (KD); myocardial dysfunction (by echocardiogram and/or elevated troponin or brain natriuretic peptide (BNP)); arrhythmia, acute respiratory failure requiring noninvasive or invasive ventilation, acute kidney injury, serositis, hepatitis or hepatomegaly, encephalopathy, seizures, coma, or meningoencephalitis together with elevated inflammatory markers.

The exclusion criteria for participants in the reviewed studies were subjects over 21 years of age, patients who were hospitalized for other conditions rather than COVID-19 and/or MIS-C, and the presence of clinically relevant bleeding within 72 hours before enrollment, as well as previous administration of antithrombotic drugs.

Types of intervention: This systematic review focuses on thromboprophylaxis in MIS-C and/or COVID-19, which is described by the administration of an antithrombotic drug, whether it is an antiplatelet or anticoagulant, and is used for the prevention of thrombotic events. We aim to review different thromboprophylaxis protocols, comparing drugs used, doses, and duration of the intervention to identify the protocol with the least thrombotic events and mortality rate.

Types of studies: We systematically examined pertinent research papers published in English and Spanish from the year 2020 to the present. We evaluated studies meeting our inclusion criteria: original quantitative studies including RCTs, cohort studies, case-control, and cross-sectional studies reporting the administration and/or consideration of administration of thromboprophylaxis as well as the thrombotic events in the population studied, coagulation times, D-dimer levels and their inflammatory markers (at least C-reactive protein).

These studies were required to report population age, an antithrombotic drug administered compared to a placebo, patient history of COVID-19 infection, thrombotic events during the study, and the parameters for the diagnosis of MIS-C. To ensure research quality, case reports, systematic reviews, narrative reviews, and meta-analyses were not included. Studies involving other populations and interventions were excluded.

Type of outcomes: The primary outcome of this review is to evaluate the efficacy of thromboprophylaxis in pediatric patients with MIS-C compared to placebo or no prophylaxis in reducing thrombotic events (PE, DVT). Secondary outcomes include mortality, clinical recovery, report of bleeding events, and report of thrombotic events. Furthermore, we will review variables such as D-dimer, coagulation times, and inflammatory markers (such as C-reactive protein) across the studies to correlate clinical reports of thrombotic or bleeding events with objective parameters. Finally, the thromboprophylactic protocols will be compared to evaluate which had the least reports of thrombotic events and mortality rates. Any other articles that did not accomplish the outcomes explained above were excluded, since they are beyond the scope of this review.

Selection of Studies, Data Extraction, and Screening

Two reviewers (NNM and CPAG) employed Rayyan (Qatar Computing Research Institute, Qatar) [8] to screen titles and abstracts. Subsequently, a third independent reviewer (AMS) verified the relevance of the studies according to predefined inclusion and exclusion criteria. Following this, a detailed full-text analysis was performed, where two other reviewers (DMML and JMCC) independently selected trials based on the same inclusion and exclusion criteria. Disagreements in this stage were similarly resolved through consensus and with the assistance of the third review author (JLC). The studies retrieved during the searches will be screened for relevance, and those identified as being potentially eligible will be fully assessed against the inclusion/exclusion criteria and selected or rejected as appropriate.

Data Evaluation: Assessment of Risk of Bias

Our evaluation followed the criteria outlined in the Cochrane Handbook [9]. The Newcastle-Ottawa Scale (NOS) [10] was used for case-control studies and cohort studies. Two independent reviewers assessed the risk of bias in each study (GVMS and KAG), considering the specific criteria and guidelines of the respective tools. Discrepancies between reviewers were resolved through discussion with a third, blinded reviewer (JLC). According to the Cochrane Handbook for Systematic Reviews of Interventions and NOS guidelines, the methodological aspects of the cohort and case-control studies were categorized as having a low, high, or unclear risk of bias. Details regarding any changes in the quality of evidence, either downgrading or upgrading, were transparently presented in the summary of findings table, along with explanations for each bias assessment.

Results

We conducted a systematic literature review to review and compare various thromboprophylaxis protocols, focusing on the specific anticoagulant drugs utilized, their dosing regimens, and the duration of interventions. The goal is to identify the protocol that most effectively minimizes thrombotic events and reduces mortality rates by analyzing a broad range of studies, including RCTs and observational data. Specifically, we focused on establishing clear, evidence-based guidelines for optimal thromboprophylaxis in clinical practice. Our search, covering the period from 2020 to the present, encompassed databases such as Pubmed, Cochrane, and Science Direct; we utilized a combination of keywords, including "Pediatric", "COVID-19", "SARS-CoV-2", "Multisystem Inflammatory Syndrome in Children", "MIS-C", "Thromboprophylaxis", "Anticoagulation", "Thrombotic events", "Prevention", "Mortality", and "Efficacy".

The cornerstone of a systematic review is the meticulous identification and selection of relevant studies from a broad literature base. Our search strategy was initiated with a comprehensive database query, resulting in 375 articles. After removing 42 duplicates, 333 unique articles were subjected to title and abstract screening, leading to the exclusion of 226 articles that did not meet the inclusion criteria. This process earmarked seven publications for full-text evaluation. Of these, three were excluded - two due to the inability to retrieve full reports and one due to the study type being inappropriate. Ultimately, this rigorous screening process refined our selection to three high-quality studies that met our stringent inclusion criteria for synthesis.

**Figure 1:**
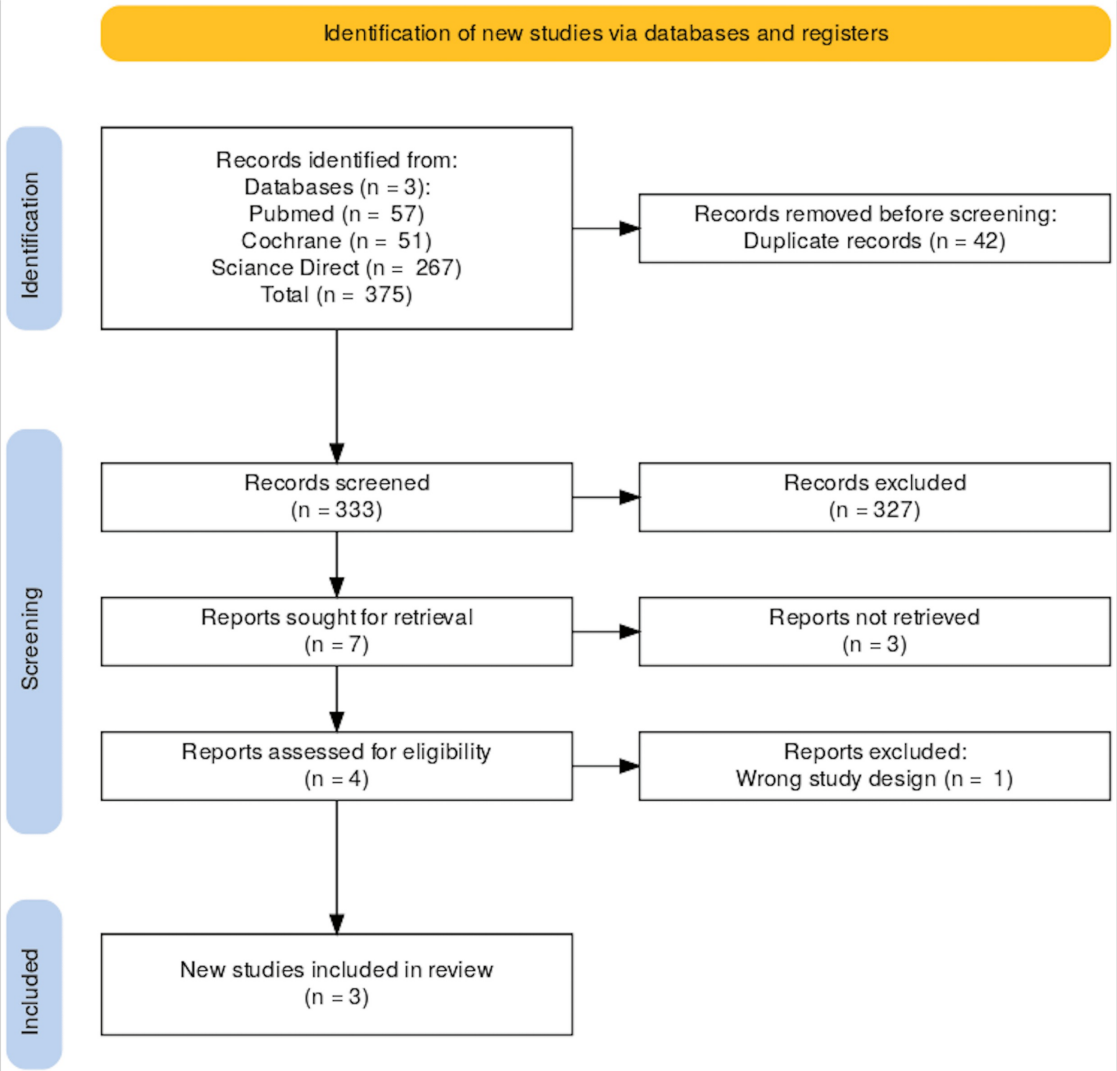
The Preferred Reporting Items for Systematic Reviews (PRISMA) Flow Diagram The PRISMA flow diagram illustrating the study selection process for the systematic review. Initially, 750 records were identified through database searches (PubMed, Cochrane, Science Direct) and registers. After removing 42 duplicates, 333 records were screened, with 326 excluded at this stage. Of the seven reports sought for retrieval, three were not retrieved. Four reports were assessed for eligibility, with one excluded due to incorrect study design, resulting in three studies being included in the final review.

Figure 1 succinctly visualizes the study selection methodology, following the design of the PRISMA flow diagram [6]. This stepwise illustration provides transparency in our filtering process, which guided the final study selection.

We conducted a thorough quality assessment of the included studies using a standardized evaluation framework. The study by Del Borrello (2020) [11] demonstrated strong methodological rigor, earning a score of 3 in the selection domain, reflecting robust participant selection procedures. The study achieved a score of 2 for comparability, indicating moderate control of confounding variables, and a score of 3 in the outcome/exposure domain, denoting a high quality of outcome and exposure assessment. With a total score of 8, this study was categorized as good quality.

Similarly, the study by Ozenen et al. (2023) [12], also a cohort design, scored 3 in the selection domain, suggesting adequate methods for participant selection. It received a score of 2 for comparability, indicating some control over confounding factors, though with potential for improvement. The outcome/exposure domain was rated 2, suggesting moderate reliability in the measurement of outcomes and exposures. The overall score of 7 placed this study in the good quality category.

The study by Schmitz et al. (2022) [5] received the highest score in the selection domain, with a score of 4, indicating excellent participant recruitment and selection criteria. However, it was rated 1 for comparability, reflecting minimal control over confounding factors. Despite this, the study scored 3 in the outcome/exposure domain, demonstrating a high level of accuracy in outcome measurement. With a total score of 8, this study was also classified as good quality.

Overall, all three studies were assessed as good quality, with total scores ranging from 7 to 8. These ratings indicate that the studies are reliable sources of evidence and contribute meaningfully to the synthesis of findings in this systematic review (Table 2).

**Table 2:** Newcastle-Ottawa Scale The studies were evaluated using the Newcastle-Ottawa Scale (NOS) [10], which assesses three domains: selection (0-4 points), comparability (0-2 points), and outcome/exposure (0-3 points). Scores range from 0 to 9, with higher scores indicating better quality. Del Borrello et al. (2020) received 8 points, Ozenen et al. (2023) received 7 points, and Schmitz et al. (2022) received 8 points. All three studies, which are cohort studies, were deemed to be of good quality.

Authors, year	Study design	Selection	Comparability	Outcome/exposure	Total	Subjective evaluation
Del Borrello et al., 2020 [11]	Cohort study	3	2	3	8	Good quality
Ozenen et al., 2023 [12]	Cohort study	3	2	2	7	Good quality
Schmitz et al., 2022 [5]	Cohort study	4	1	3	8	Good quality

The reviewed studies consistently highlight the varied incidence of thromboembolism among pediatric patients with COVID-19 and MIS-C. Del Borrello et al. (2020) [11] reported no thrombotic events among 35 hospitalized pediatric patients, despite elevated D-dimer levels in both COVID-19 and MIS-C cases, with higher levels observed in the latter. This absence of thromboembolic complications may suggest the effectiveness of targeted thromboprophylaxis in this cohort. In contrast, Ozenen et al. (2023) [12] observed thrombotic events in 0.4% of a larger cohort of 690 pediatric patients, with a higher incidence in MIS-C patients (2.1%) compared to those with COVID-19 (0.2%). This finding indicates that while thromboembolism is rare, MIS-C patients may be at a higher risk, underscoring the need for vigilant monitoring. Schmitz et al. (2022) [5] further support this, reporting a 2.2% incidence of VTE in their cohort of 211 hospitalized pediatric patients, suggesting that even with thromboprophylaxis, a small but significant risk of VTE remains, particularly in MIS-C patients.

The thromboprophylaxis protocols varied across the studies, with different drugs, dosing regimens, and durations used. Del Borrello et al. (2020) [11] primarily used enoxaparin (100 U/kg every 24 hours) and occasionally unfractionated heparin (10 U/kg/h) for patients with higher bleeding risks, continuing prophylaxis until discharge or until thrombotic risk factors were resolved. Ozenen et al. (2023) [12] employed both enoxaparin and aspirin, with 69.1% of MIS-C patients receiving enoxaparin for a median of 9.5 to 10 days and 90.4% receiving aspirin for 30 to 42 days. This study highlights a more aggressive prophylactic approach in MIS-C patients, reflecting the higher thrombotic risk in this group. Schmitz et al. (2022) [5] used a tailored-intensity approach, with 45 patients receiving either prophylactic or therapeutic doses of anticoagulation based on their VTE risk factors and disease severity, adjusting the duration of thromboprophylaxis according to the normalization of D-dimer levels, reflecting a more dynamic approach to treatment.

Outcomes associated with thromboprophylaxis varied slightly among the studies. Del Borrello et al. (2020) [11] reported no thrombotic events or deaths, suggesting that their tailored approach was effective in preventing thromboembolism without increasing bleeding risk. Similarly, Ozenen et al. (2023) [12] found no mortality, though 17.5% of patients on aspirin developed elevated transaminases, indicating a need for careful monitoring of liver function during prolonged antiplatelet therapy. Despite the rare occurrence of thrombotic events, the study suggests that prophylaxis may still be warranted in high-risk patients. Schmitz et al. (2022) [5] reported one case of VTE and one unrelated death, with no major bleeding events observed, though minor bleeding occurred in seven patients. This study underscores the safety of tailored-intensity thromboprophylaxis, balancing the prevention of thrombotic events against the risk of bleeding.

In comparing the three studies, a consensus emerges on the importance of thromboprophylaxis in preventing thromboembolic events in pediatric COVID-19 and MIS-C patients. However, the approach to thromboprophylaxis varies significantly. Del Borrello et al. [11] advocate for a more selective use of anticoagulants, guided by individual risk factors rather than routine D-dimer values. Ozenen et al. [5] and Schmitz et al. [12], however, emphasize the need for a more aggressive and tailored approach, particularly in MIS-C patients who are at higher risk of thromboembolism. The use of enoxaparin and aspirin as primary agents across studies highlights their importance in pediatric thromboprophylaxis. The variable dosing regimens and durations suggest that a one-size-fits-all approach may not be optimal and that treatment should be individualized based on patient risk factors and response to therapy.

These findings support the development of standardized, evidence-based guidelines for thromboprophylaxis in pediatric patients with COVID-19 and MIS-C, with a focus on balancing efficacy in preventing thromboembolism against the potential risks of bleeding and other adverse events. Further research is needed to refine these protocols and identify the most effective and safest strategies for this vulnerable population (Table 3).

**Table 3:** General Outcomes Summary VTE: venous thromboembolism; LMWH: low molecular weight heparin; UFH: unfractionated heparin; HIT: heparin-induced thrombocytopenia; BID: bid in die (twice a day); BMI: body mass index; BQM: Braden Q mobility score; CNS: central nervous system; CVC: central venous catheter; EF: ejection fraction; IBD: inflammatory bowel disease; LIC: localized intravascular coagulation; nt CICC: non-tunnelled centrally inserted central catheter; PICC: peripherally inserted central catheter; PICU: pediatric intensive care unit; t CICC: tunnelled centrally inserted central catheter; TPN: total parenteral nutrition; SLE: systemic lupus erythematosus

Study ID	Author and the year of publication	Country	Study design	Total sample size	Mean age ± SD	Criteria considered for thromboprophylaxis administration	Thromboprophylactic drug and dosage	Comparison	Duration of intervention	Thrombotic events and mortality rate with/without intervention	Coagulation times before and after intervention or with different interventions	D-dimer levels (ng/mL)	Inflammatory markers (e.g. C reactive protein)	Bleeding events	Keynotes
										Before and after intervention or with different interventions					
1	Del Borrello et al. (2020) [11]	Italy	Cohort study	36 COVID-19 = 30; MIS-C = 6; Prophylaxis = 6	COVID-19 (median age 3 years, range 10 days to 19 years); MIS-C (median age 6.8 years, range 4.5 to 12.5 years)	The Institutional Risk Assessment Model (RAM) assigns initial scores to patients based on disease severity: +2 points for MIS-C and +1 points for COVID-19 in cases of at least "moderate" severity. Key factors considered in the scoring include age, mobility, presence of a central venous catheter (CVC), infections, thrombophilia, trauma or surgery, admission to the PICU, cardiovascular comorbidities, and bleeding risks. If the total score reaches 3 points or more, and there are no bleeding risks, prophylactic anticoagulation and a hematology consultation are recommended.	100 U/kg every 24 hours (n = 4); heparin (UFH) at 10 U/kg/h (n= 2)	No thromboprophylaxis administration.	Until discharge or thrombotic risk factor resolution/attenuation.	With intervention: None; Without intervention: None	PT ratio baseline= 1.1; PCM = 1.2; Resolution= 1.1	Mildly affected (n=14), baseline = 814, moderately to critically ill (n=10), baseline = 916. Peak of clinical manifestations (PCM) = 1200; Resolution = 416; Severely/critically (n=6); Baseline = 823; MIS-C (n=6) Baseline average = 1900	Moderately affected to severe (n=22); Fibrinogen: Baseline = 373; PCM = 348; Resolution= 233; CPR Baseline= 9; PCM = 26; Resolution= 1; Platelets (x 10^9/L); Baseline = 223; PCM = 225; Resolution = 292	None	In contrast to adults, this state is unlikely to lead to clinically significant thrombotic complications in children. This study does not recommend universal anticoagulant prophylaxis for hospitalized children with COVID-19, except in selected cases with multiple pro-thrombotic risk factors (obesity, active malignancy, or sickle cell disease), without relying solely on D-dimer levels (they primarily indicate an acute-phase response). For these patients, a personalized prophylactic anticoagulation (PA) strategy, possibly using enoxaparin or unfractionated heparin (UFH), may be considered. High inflammatory markers are better suggestive of MIS-C.
2	Ozenen et al. (2023) [12]	France	Single-center retrospective study	690 COVID-19 = 596; MIS-C= 94; Prophylaxis= 154	COVID-19 (median 64.5 months, range 20 days to 17 years and 6 months); MIS-C (median 83.5 months, range 5 years to 17 years)	Risk factors for VTE include obesity, immobility, extended hospitalization, congenital heart disease, a prior history of VTE, and/or significantly elevated D-dimer levels (greater than five times the normal upper limit) and/or severe illness.	Enoxaparin 1x100 IU/kg and 2x100 IU/kg (median 10 days, range 1 day to 180 days). Aspirin 80 mg/kg for 3-5 days then 3-5 mg/kg (median 30 days, range 2 days to 180 days)	Antithrombotic prophylaxis with enoxaparin: COVID-19 during hospitalization (15.9%); on discharge (34.9%). MIS-C during hospitalization (71.4%); on discharge (2.2%). Antithrombotic therapy with aspirin: COVID-19 during hospitalization (12.7%); on discharge (12.7%). MIS-C during hospitalization (28.6%); on discharge (28.6%)	The overall median hospital stay for all patients was 5 days (ranging from 1 to 56 days), with a statistically significant longer duration observed in the MIS-C group (p < 0.001).	Thrombosis was observed in one (0.2%) patient in the COVID-19 group and in two(2.1%) patients in the MIS-C group. With intervention: A 13-year-old patient received prophylactic therapy for one month. Without intervention: One of them was a 14-year-old girl diagnosed with mild COVID-19 and a 16-year-old boy diagnosed with mild MIS-C.	Before intervention: PT (median 13-sec range 8.5 sec to 38.1 sec); APTT (median 31.1 sec, range 16.6 sec to 73.7 sec); INR (median 1.1 sec, range 0.4 sec to 2.0 sec). After intervention: PT (median 13.4 sec, range 9.8 sec to 21.9 sec). APTT (median 30.0 sec, range 17.2 sec to 44.5 sec); INR (median 1.18 sec, range 0.8 sec to 1.8 sec)	Before intervention: Median 225 range 41 to 13333. After intervention prophylaxis antithrombotic Median 563 ranges from 150 to 9924	Before intervention. CPR: median 0.4 range 0.02 to 35.7; COVID-19 median 0.2, range 0.02 to 22.4; MIS-C median 13.0 range 0.02 to 35.7. WBC: median 7.3 range 1.1 to 7.4; COVID-19 median 6.6, range 1.1 to 47.4; MIS-C median 10.3, range 1.0 to 26.1; Fibrinogen: median 316 range, 37 to 3030; COVID-19 median 294, range 37 to 3030; MIS-C median 593, range 277 to 1496. After intervention. CPR: median 6.9, range 0.02 to 35.7 COVID-19 median 5.2, range 0.2 to 32.3; MIS-C median 0.2, range 0.02 to 22.4; WBC: median 7.8, range 1.0 to 26. COVID-19 median 10.8, range 2.1 to 23.8; MIS-C median 6.7, 1.1 to 47.4 Fibrinogen: median 486, range 161 to 1496 COVID-19 median 533, range 271 to 823 MIS-C median 279, range 37 to 3030	None	This study aimed to assess the incidence of thrombotic events in pediatric patients with COVID-19 and MIS-C and to analyze laboratory findings before and after the use of antithrombotic prophylaxis. Antithrombotic prophylaxis was administered to patients with risk factors for VTE, such as obesity, immobility, prolonged hospitalization, congenital heart disease, and a prior history of VTE. The findings revealed that no thrombotic events occurred in children with risk factors or underlying conditions who received prophylaxis. Notably, no patient in the study died, although 44 required admission to the PICU. Regarding thrombotic events, only three cases were documented: two occurred in patients who did not receive antithrombotic prophylaxis, and one was reported in a patient who developed thrombosis after one month of aspirin therapy.
3	Schmitz et al. (2022) [5]	USA	Single-center observational cohort study	Total = 45; COVID-19 = 16; MIS-C = 29	14.8 years	Moderate (WHO progression scale 4-5) to severe (WHO progression scale 7-9) disease and exposure to >1 risk factor for VTE; no overt risk of bleeding. Titration to therapeutic dosage in case of underachievement of anti-Xa levels or respiratory failure/hemodynamic instability.	Prophylactic intensity: LMWH 0.5 mg/kg/dose BID OR UFH 10-15 Units/kg/hour (24, 53.3%)	Therapeutic intensity: LMWH 1 mg/kg/dose BID OR UFH 20 Unit/kg/hour (21, 46.7%)	Median: MIS-C, 22 days; COVID-19, 19 days. Normalization of D-dimer values.	Prophylactic dose: 1 patient with VTE. No pulmonary embolism or fatal VTE. Therapeutic dose: No VTE, pulmonary embolism, or fatal VTE. All-cause mortality 2.2% (95% CI, 0.06%-11.8%) unrelated to bleeding/thrombosis.	Prothrombin Time(s): 12.5 (12.0-13.0) Partial thromboplastin time(s) 29.5 (27-31)	At presentation: 2.9 (21.9-3.8) At discontinuation: 0.28 (0.23-0.39)	CRP: 11.8 mg/dl (4.7-18.1); COVID-19, 4.1 mg/dl; MIS-C, 14.6 mg/dl. Ferritin: 445 ng/ml (267-890) COVID-19, 7.42 ng/ml; MIS-C, 42 ng/ml. NT-proBNP: 1628 pg/ml (210-4450) COVID-19, 100 pg/ml; MIS-C, 2930 pg/ml.	Major bleeding: 0 (0%). Minor bleeding events: 7 (15.5%)	This study suggests that monitoring D-dimer levels at three-week intervals may assist in determining when to discontinue anticoagulation therapy in MIS-C patients. D-dimer levels correlated with the disease progression, showing elevation at presentation due to SARS-CoV-2-induced inflammation and a decrease or normalization as inflammation subsided within 2-3 weeks. Median blood counts and coagulation screening were performed for most patients and were generally within normal ranges. Inflammatory markers, such as C-reactive protein (CRP) and ferritin, were significantly elevated in the MIS-C cohort compared to those with COVID-19. Risk factors for VTE included obesity, the presence of a central venous line (CVL), invasive ventilation, severe dehydration, personal or family history of thrombophilia, immobilization for 48 hours or more, estrogen-containing hormonal therapy, pregnancy, cancer, asparaginase therapy, major trauma, and inflammatory conditions. MIS-C was explicitly recognized as an inflammatory risk factor. For assessing severity in MIS-C patients, the WHO progression scale was adapted: patients receiving care on an acute floor were classified as "moderate," while those requiring ICU admission with ventilator or vasopressor support were categorized as "severe."

Discussion

The objective of this systematic review has been to identify studies that report the association between COVID-19, or MIS-C, thromboembolism as well as the effects of thromboprophylaxis on these events, particularly in pediatric patients. We have found three articles where we reviewed the protocols applied in each one, comparing the medications, their doses, and duration of treatment, trying to identify the protocol with the lowest number of thrombotic events and mortality rate.

Del Borrello et al.'s [11] cohort study involves 36 patients, of whom only six received prophylaxis due to a diagnosis of MIS-C. The study reports no deaths but does not specify the duration of anticoagulant therapy. Enoxaparin and unfractionated heparin were used until the thrombotic risk factors were resolved or diminished.

In Ozenen et al.'s [12] study, they managed a larger cohort of up to 690 patients, of which 154 received prophylaxis with enoxaparin and aspirin. They also had no deaths, and the median duration of treatment with enoxaparin was 10 days and with aspirin 42 days, reporting the presence of thrombosis in three of their patients, being previously healthy patients, corresponding to a frequency rate of 3.3%; none of the two patients were receiving prior thromboprophylaxis, and only one of them was receiving treatment with aspirin for one month of evolution due to having been hospitalized with MIS-C previously in another hospital. Although obesity is among the risk factors for the presence of thrombotic events, this review shows that the reported thrombotic events occurred in previously healthy patients.

In Schmitz et al.'s [5] study, they had 45 patients, of whom 24 received therapeutic doses and 21 prophylactic doses, 23 in concurrent treatment with aspirin for thromboprophylaxis; three received initial treatment with unfractionated heparin and ongoing monitoring to monitor anti-Xa levels, reaching goals within the first 48 hours. The mean dose for low molecular weight heparin for both prophylaxis and therapeutics was 0.5 mg/kg/do and 1 mg/kg/do twice daily, and the mean duration of anticoagulation was 19 days. Its mortality rate was 2.2%, with a single death reported within three months after discharge.

In an Italian observational study by Del Borrello et al. [11], the prevalence of VTE was recorded as 1 in every 350 pediatric patients hospitalized for COVID-19, which is higher than the rate reported in the general pediatric inpatient population (1 in every 200 patients).

With the above data, we can emphasize the high rate of effectiveness in reducing or eliminating the presence of thrombotic events with thromboprophylaxis, regardless of the scheme used. Although the anticoagulation schemes reported are variable, they agree that they should only be used or applied in cases of high risk of thrombosis, demonstrating that each case must be evaluated and individualized.

The studies analyzed in this review have shown risk factors associated with thrombosis, which help us establish thromboprophylaxis only in necessary cases. We consider it of vital importance that they have included patients with comorbidities such as hematological and erythrocyte diseases; however, there are still few patients studied in this regard, the rest of the cohorts studied are a large volume of patients but previously healthy, highlighting that the fact of being healthy does not exclude them from presenting thrombotic events if they are not treated with adequate thromboprophylaxis. Another point in favor of these studies is knowing the admission to intensive care units, which is a measure or index to evaluate the severity of the disease in patients.

Monitoring for anticoagulation is an important point that should be evaluated in subsequent studies, since it is the only way in which we can determine the end of the proinflammatory state of the disease by monitoring D-dimer levels. From now on, to think in subsequent studies, we could perform D-dimer measurements prospectively to determine the duration of treatment in each patient more specifically, since even if they are discharged, the inflammatory risk state may persist. Patients with MIS-C are those who should always be on thromboprophylaxis, given the high risk of thrombosis.

## Conclusions

This systematic review confirms that pediatric patients with COVID-19 or MIS-C face a significant risk of thrombotic events, particularly due to the proinflammatory state in MIS-C. Thromboprophylaxis has proven effective in reducing these events, especially when initiated in patients with risk factors such as obesity, prolonged immobilization, and severe inflammation. Enoxaparin and aspirin were commonly used across studies, with protocols tailored to patient risk and D-dimer levels. The reviewed evidence supports thromboprophylaxis in high-risk pediatric cases, emphasizing the importance of individualized treatment to balance efficacy and bleeding risks. Further research is needed to optimize treatment duration and develop standardized guidelines for preventing thrombotic events in this population.
